# Editorial: Palliative and end-of-life care in the post-pandemic era: old problems and new perspectives

**DOI:** 10.3389/fsoc.2025.1679984

**Published:** 2025-09-03

**Authors:** Barbara Sena, Enrico De Luca, Guido Giarelli

**Affiliations:** ^1^Department of Letters, Philosophy, Communication, University of Bergamo, Bergamo, Italy; ^2^Department of Nursing and Midwifery, University of Birmingham, Birmingham, United Kingdom; ^3^Department of Health Sciences, University ‘Magna Graecia' of Catanzaro, Catanzaro, Italy

**Keywords:** end-of-life care, palliative care, post-COVID, health services, health policies and management

The problem of prolonging life or accompanying death is not new in the fields of social science and healthcare and has been a subject of discussion for many years. It remains one of the most emblematic issues in modern medical science, which (Illich 1976) defined as the “medicalisation of dying,” highlighting its impact on how death is approached in modern culture. Sociological interest in this topic began with the work of (Glaser and Strauss 1965) on awareness of dying and continues today with studies on the challenges of managing dying patients in various care settings (e.g., Sudnow, 1967; Broom, 2015; Bandini, 2020; Sena and De Luca, 2022).

In this context, palliative care (initially known as “terminal care”) has become synonymous with end-of-life management. It can be considered a *postmodern* specialty because it lacks a specific disease, bodily organ, or life stage to call its own. Therefore, it continues to be subject to prejudice, which relegates the role of palliative care to only treating pain in dying patients (Masel and Kreye, 2018).

During the most critical phases of the COVID-19 pandemic, managing the end-of-life became exceedingly complex, especially in emergency wards and intensive care units. This complexity extended even to countries and regions that had previously experienced high mortality rates and whose healthcare workers were well-versed in providing compassionate palliative care, despite resource limitations and during humanitarian crises. However, the significant global rise in COVID-19-related deaths further intensified and complicated the delivery of end-of-life and palliative care, requiring innovative approaches beyond existing ones. Many healthcare professionals were unprepared to navigate the ethical dilemmas between proper treatment and a dignified death (Nicoli and Gasparetto, 2020). As healthcare resources and facilities faced unprecedented pressure, valuable lessons could be learned from models of care in other settings around the world.

Three years after the pandemic, the field of end-of-life and palliative care remains underexplored in health sociology. This Research Topic addresses this gap with seven contributions.

The first article, by Clancy et al., presented a co-produced Creative Toolkit© to support the wellbeing of palliative care professionals, especially in relation to COVID-19-related stress. This arts-based resource, incorporating music, visuals, and theater, addressed holistic needs. Organizations such as Hospiscare UK have reported emotional benefits and stronger team bonds as a result of using the toolkit. The study aligns with wider evidence on the role of art in mental health and calls for its broader implementation.

The second article, by Hodge et al., examined the barriers to palliative care in the South-West of England—a region that is facing aging and rural healthcare challenges. Through 13 focus groups, the authors identified issues such as poor out-of-hours services, fragmented communication, and discomfort around death. The authors called for more coordinated, person-centered practices that reflect patient values and support equitable, compassionate end-of-life experiences.

Next, Zhang et al. analyzed the attitudes toward palliative care of 541 cancer patients in China, using a multi-method approach. Despite its benefits, acceptance was low. Education, occupation, caregiving experience, insurance, cancer stage, and anxiety influenced perceptions. From a sociological perspective, the study shows how culture and inequality shape understanding and stresses the need for education and culturally sensitive approaches.

Juan et al. addressed Singapore's underuse of palliative services, attributing it to limited provider knowledge. Through a nationwide cross-sectional online survey conducted among primary and tertiary healthcare providers, the authors assessed the challenges they face, their palliative education, their confidence in managing patients undergoing palliative care, and their knowledge of palliative surgery. Their findings confirm that healthcare providers in Singapore have poor knowledge of, and misconceptions about, palliative care and surgery, making it essential to improve awareness and education among those caring for seriously ill adults.

Barasteh et al. explored Iran's palliative care landscape through a three-phase qualitative study (2018–2020), outlining future scenarios up to 2030. Their results show that the development of palliative care within the Iranian healthcare system faces serious uncertainties and that palliative care development efforts need to focus on two axes: social acceptance and the need for consistent governance by the Ministry of Health.

Vitorino et al. reflected on how the disruption caused by the COVID-19 pandemic to end-of-life care highlighted the importance of strengthening community support networks (comprising family, friends, neighbors, and community members) as the foundation of compassionate community efforts to enhance their capacity to care for others and improve the overall experience of death, including the process of dying and the ensuing bereavement period. Therefore, active community participation and death education can strengthen a community's capacity to assist those coping with death, dying, and bereavement.

The latest article written by De Luca et al. introduced the final article introduced spiritual care models in palliative care. While Western healthcare increasingly recognizes the importance of spirituality, its integration into daily practice remains rare. Palliative care, however, has begun to address the psychological and existential dimensions of care through holistic models that transcend biomedical frameworks. This concept analysis examines the challenges faced by clinicians when implementing shared, patient-centered spiritual practices. Drawing on case studies from Thailand and Italy, and introducing two UK-based conceptual models, this analysis advocates for shared, patient-centered spiritual practices and assessments These models enable meaningful dialogue, enhancing the therapeutic relationship and fostering compassionate, person-centered care.

The variety of contexts, approaches and issues addressed in these articles demonstrates the growing attention being given to end-of-life care and management issues in Western and Eastern contexts alike. The aim of this Research Topic was therefore to draw the attention of medical sociology scholars to this topic, in the hope of encouraging further research.
